# Play Well Triple P: Developing and Evaluating a Program to Promote Positive Parental Involvement in Junior Sport

**DOI:** 10.1007/s10578-024-01725-y

**Published:** 2024-06-18

**Authors:** Cassandra K. Dittman, Matthew R. Sanders, Steven B. Rynne, Clifford J. Mallett, Jordan S. Lefebvere

**Affiliations:** 1 Cluster for Resilience and Wellbeing, Appleton Institute, Central Queensland University, Bundaberg, Australia; 2 Manna Institute, Central Queensland University, Bundaberg, Australia; 3 School of Psychology, The University of Queensland, Brisbane, Australia; 4 School of Human Movement Studies and Nutrition Sciences, The University of Queensland, Brisbane, Australia; 5 School of Health, Medical and Applied Sciences, CQUniversity, Locked Bag 3333, Bundaberg DC, QLD 4670 Australia

**Keywords:** Parenting, Parenting intervention, Parenting program, Sport, Evaluation, Sports parenting

## Abstract

Parents play a central role in children’s ongoing participation and enjoyment of sport. Despite compelling evidence that parental behaviour can undermine the quality of children’s experiences in sport, little research has examined whether strategies to support parents to engage in positive sports parenting behaviour are effective. In this paper, we report two studies that had the overall aim of developing, implementing and evaluating a program designed to promote positive parental involvement and improve spectator behaviour in junior rugby league in Australia. Study 1 involved the development of a prototype version of the program, named Play Well Triple P, which was qualitatively evaluated through interviews with 19 parents about their satisfaction with the program. In Study 2, we used feedback from Study 1 to refine the program and develop a pilot version, which was then evaluated in a quasi-experimental feasibility study with 101 parents (mean age = 38.42 years; 72% mothers) of junior rugby league players. The pilot version involved one interactive online module and text messages to reinforce content and prompt strategy implementation across the season. Participation in Play Well Triple P was associated with increased positive sports parenting behaviour and reduced controlling and intrusive sports parenting behaviour, with a trend towards reducing over-reactive parenting practices at home. These findings are discussed in relation to the feasibility of implementing a brief and engaging sports parenting intervention in the context of a broader integrated system designed to facilitate ongoing participation of children in sport.

**Supplementary Information:**

The online version contains supplementary material available at 10.1007/s10578-024-01725-y.

## Introduction

Participation in organised sport is associated with many physical, social, and psychological benefits for children and adolescents. As well as promoting physical health and fitness (Telford et al. 2016), organised sport provides a context to build a wide range of positive developmental skills and competencies in children and adolescents, including self-esteem, emotional regulation skills, resilience, and better social relationships (Eime et al. 2013). Relatedly, organised sport promotes young people’s wellbeing (Wilson et al. 2022), and plays a protective role in preventing mental health issues such as anxiety, depression, and suicidality (Graupensperger et al. 2021; Vella et al. 2015). These positive psychological and mental health outcomes appear to be even more apparent when children and adolescents participate in team sports (Pluhar et al. 2019; Zuckerman et al. 2021). Given the importance of sport for maintaining children’s social and psychological wellbeing, research is needed to identify strategies for supporting children to retain positive involvement in sport throughout childhood and adolescence. The present study focuses on the influence of parental behaviour and examines the efficacy of a brief intervention on promoting positive and constructive parental involvement in children’s sport.

Parental behaviour has been highlighted as an important interpersonal factor influencing children’s experience and ongoing participation in organised sport (Crane and Temple 2015; Moulds et al. 2022). Beyond the important logistical and financial support parents provide, they also facilitate their children’s enjoyment of and adaptive motivation in sport through emotional support and encouragement, unconditional acceptance, and praise and reinforcement, particularly when it is linked to improvement and skill development (Dorsch et al. 2021). For instance, a study with 317 adolescent English football players found that parental praise and understanding was associated with adolescents’ enjoyment of football (Mossman and Cronin 2019), while research with 380 secondary school students and their parents in the United States found that parental support influenced young people’s sport participation directly, and indirectly through a positive influence on adolescent’s self-efficacy in sport (Trost et al. 2003). These findings are consistent with qualitative research in which children and adolescents have reported that receiving emotional support and encouragement from their parents helps them feel motivated and gain enjoyment from sport (Furusa et al. 2021; Knight et al. 2011; Strandbu et al. 2019).

While most parents’ intentions are to support and encourage children, parental expectations, attitudes, and behaviours can unintentionally have a negative impact on children’s sporting experience. Specifically, parental pressure characterised by unrealistically high expectations, a focus on winning and/or outperforming peers, and harsh comments, alongside directive and controlling parenting practices, have been identified across several studies as having negative implications for children’s sporting experience (Dorsch et al. 2021). Parental pressure and directive sports parenting practices have been found to be related to negative child sporting outcomes, including lower motivation (Amado et al. 2015; Sánchez-Miguel et al. 2013), lower enjoyment (Amado et al. 2015; Ryan Dunn et al. 2016; Sánchez-Miguel et al. 2013), higher competition anxiety (Bois et al. 2009; O’Rourke et al. 2011), and sport dropout (Crane and Temple 2015). Qualitative research has elucidated the parental behaviours that children and adolescents perceive as being detrimental to their enjoyment and motivation in sport (Elliott and Drummond 2017a, b; Knight et al. 2011; Omli and Wiese-Bjornstal 2011; Tamminen et al. 2017). The young people in this body of research identified problematic parental behaviours that occur before, during and after sporting events, including publicly given commands and criticism, derogation of opposition players, arguing with referees or coaches, angry outbursts, excessive cheering and challenging ‘debriefing’ conversations on the drive home.

Overall, there is compelling evidence that parental behaviour has important implications for children’s enjoyment and ongoing participation in sport. A recent meta-synthesis of 58 qualitative studies highlighted the strong emotional reactions parents experience in response to sports-related events, along with the learning process parents go through to understand how to be respectful spectators (Sutcliffe et al. 2021). Other research has highlighted that parents’ historical and social context, such as their personal sporting history (Knight et al. 2016) and social identification with their children’s sport (Mallett et al. 2024), influences their sports parenting expectations and behaviour. Yet, most parents receive little to no preparation or skills training in how to support their children’s participation in sport, including appropriate spectator behaviour (Knight et al. 2017). Internationally, most junior sporting organisations incorporate strategies, policies, or practices to manage negative spectator behaviour, such as codes of conduct, parent communication campaigns, and parent liaison officers. However, these global, indirect approaches are not always evidence-based, have not been properly evaluated, and appear to be doing little to address poor parental behaviour in junior sport (Elliott and Drummond 2015).

Clearly, targeted and bespoke programs are needed that support parents to engage in behaviours that facilitate positive involvement in their children’s sport (Sanders et al. 2024). A vast body of evidence has shown that parenting programs that teach parents to engage in positive and constructive parenting practices has a positive impact on children’s mental health and development (Barlow and Coren 2018; Jewell et al. 2022), with a handful of studies demonstrating the feasibility and efficacy of doing this in a junior sports context (e.g., Dowell et al. 2021; Smoll et al. 2007). Recently, several important examples of evidence-based sports parenting interventions have emerged, with evaluations of these programs supporting the feasibility and value of such programs (Burke et al. 2021). Thrower et al. (2017) qualitatively evaluated the efficacy of a 6-session, face-to-face group program for parents of tennis players. They found that parents reported improved understanding of, and confidence in, their involvement in junior tennis. Notably, Dorsch et al. (2017) conducted a controlled trial of a sports parenting intervention for 81 parents of 7- to 10-year-old soccer players. They found that parents who participated in the full intervention (i.e., 45-minute parent seminar plus sports parent guide) had children who reported more positive sports parenting (i.e., more support, and less pressure and conflict), and greater enjoyment and sense of competence in soccer in comparison to parents who received the parent guide and to parents who received no intervention.

While the results of these studies are promising, both studies highlighted the time, resource, and personnel costs typically associated with face-to-face interventions, as well as issues in sustaining parent attendance and engagement over the course of the intervention (Ingoldsby 2010). As a result, face-to-face interventions are difficult to take to scale, which is an important consideration in the design of sports parenting interventions, given the vast number of children enrolled in organised sport (Eime et al. 2016). To help address these issues, Thrower et al. (2019) adapted their parents in junior tennis program for online delivery. In a mixed methods pilot study of the 7-session program with 13 parents, quantitative results revealed a trend towards improved sports and general parental self-efficacy and decreased ego goal orientation, while qualitative findings indicated acceptance and satisfaction with the content and online delivery of the program. This suggests that web-based delivery may be a promising means of delivering a sports parenting intervention.

### The Current Studies

The overall aim of this research was to develop, implement, and evaluate a sports parenting intervention to provide parents with evidence-based strategies for enhancing constructive parental involvement and improving spectator behaviour in junior rugby league. Rugby league is a highly popular junior sport in Australia, with almost 150,000 young people aged 5 to 18 enrolled in the sport across Australia. The study employed an exploratory sequential mixed methods approach involving the collection of qualitative feedback from parents on a prototype version of the program and a pilot evaluation of the revised version of the program using quantitative measures.

The intervention strategy, *Play Well Triple P* (PWTP), aimed to be “fit for purpose” in terms of its applicability to the Australian junior rugby league context, and easily deployable in junior rugby league clubs. To facilitate accessibility, the objective was to deliver PWTP as an online program involving minimal time commitment from parents (< 2 h). The content of PWTP was based on the core parenting strategies and concepts from the evidence-based Triple P – Positive Parenting Program (readers are referred to Sanders et al. 2023 for an overview of the Triple P system and its objectives and theoretical background). Triple P is a multi-level system of parenting support involving a suite of 33 discrete interventions ranging in intensity from brief, topic-specific resources and programs designed for universal access by all parents, to more intensive small group and individual multi-session interventions for families experiencing disruptive child behaviour and other family problems. Meta-analyses confirmed the effectiveness of Triple P in its various delivery formats in reducing child behaviour problems and improving parenting skills and confidence (Sanders et al. 2014b), while controlled trials have supported the effectiveness of the online variant of Triple P (Sanders et al. 2012, 2014a). Play Well Triple P was designed as a universal program within the Triple P system suitable for all parents with children playing rugby league.

The first phase of this research involved an online survey of 1,418 parents of children playing junior rugby league (Mallett et al. 2024) to collect information regarding the frequency of problematic parenting behaviour at junior rugby league events, and to identify parental factors related to negative sports parenting behaviour that could be modified in a parenting intervention. The findings indicated that parents’ competitive attitudes, emphasis on winning, poorer emotional wellbeing, and controlling parenting practices were associated with parents reporting negative emotional reactions and inappropriate spectator behaviour at their children’s games (Mallett et al. 2024). Overall, the findings evidenced the need for a parenting support intervention in junior rugby league and identified the parenting factors to be targeted in such an intervention.

The second, third, and fourth phases of this research are reported within this paper. Phase 2 is reported in Study 1 where the aim was to collect qualitative feedback from junior rugby league parents about their satisfaction and acceptance of a prototype of PWTP. Phases 3 and 4 are reported in Study 2 where the aim was to (a) use the feedback obtained from Study 1 to refine the program and develop a pilot version for evaluating; and (b) quantitively evaluate the effects of PWTP on sports parenting behaviour and expectations.

## Study 1

### Aims

Study 1 aimed to qualitatively evaluate a prototype version of PWTP to obtain information on parental acceptability and satisfaction with the program. To achieve this, parents were asked to complete the two online PWTP modules that made up the prototype (see below for more details) and then to participate in an interview to obtain their feedback on the program.

### Method

Participants A sample of 19 parents were recruited through email and social media outreach to two junior rugby league clubs in Southeast Queensland who were nominated by the Queensland Rugby League (QRL) to be involved in the project. Recruitment and data collection occurred from August to October, coinciding with midway through the 2020 season, which had a delayed start because of COVID-19 lockdowns. Participants ranged in age from 30 to 56 years (mean age = 41.7 years, *SD* = 6.01) and were the parents of at least one child aged 6 to 12 years enrolled in the 2020 rugby league season. All but one of the parents (94.7%) volunteered in some capacity with their child’s club, with six parents (31.6%) serving as their child’s coach. Ten (52.6%) of the respondents were fathers, and nine were mothers (47.4%). Fourteen (73.7%) of the respondents identified as Australian, three as New Zealand Pākeha (15.8%), one as being from a Polynesian cultural background and one from a North-Western European background. Most parents were employed in either full- or part-time work (88.9%), and most (78.9%) had a post-secondary school qualification (47.4% vocational qualification; 31.6% university degree).

#### Procedure

The study was approved by the ethics committee of the authors’ university (approval #: 2020001179). Participants expressed their interest in participating in the study by emailing the research team. A team member then contacted participants by telephone to describe the aims of the study and the nature of their participation, assess their eligibility for the study (i.e., were a parent of at least one child aged 6 to 12 years playing junior rugby league), and describe how to access the PWTP online program. Participants were emailed a link to a Qualtrics survey to provide informed consent for the study and to provide basic demographic information about themselves and their child. At the end of the survey, participants were given a link to register for the online program.

Parents were given 2 weeks to complete the online modules before they were contacted to schedule the interview. In total, 22 parents were given access to the program, but only 19 parents completed their scheduled interview. Scheduling difficulties prevented the other 3 parents from being interviewed. Interviews were conducted during September 2020 on Zoom by a research assistant trained in interviewing, and under the supervision of two of the authors SR and CM. Interviews lasted an average 17:29 min (*SD* = 4:20 min) and were audio-recorded for later transcription.

A semi-structured interview protocol was developed based on the aims of the study. Key areas of interest were addressed using open-ended questions and follow-up prompts. Questions assessed acceptability of the program (e.g., “What did you like about Play Well Triple P?”, “Was there anything you didn’t like?”), usefulness (e.g., “Was the program helpful for you?”) and relevance (e.g., “Did you relate to the content of Play Well Triple P?”) of the content, and intention to use the strategies in the program (e.g., “Have you used any of the tips or strategies already?”, “Do you intend to use any?”). Participants were also asked about implementing the program in clubs (e.g., “How do you think the program could be delivered to parents?”).

Finally, at the end of the interview, the interviewer asked participants to rate their satisfaction with the program using the Client Satisfaction Questionnaire (CSQ), a measure routinely used in evaluations of Triple P programs. The version used in this study was adapted from the version of the CSQ developed to evaluate satisfaction with the online variants of Triple P (Sanders et al. 2012). The interviewer verbally read each of the 11 statements about program quality, relevance and usefulness to participants, who were asked to rate each statement on a 5-point scale from 1 (*Strongly Disagree*) to 5 (*Strongly Agree*). Higher ratings reflected greater satisfaction.

#### Program Description

PWTP is underpinned by a self-regulatory model of intervention (Sanders and Mazzucchelli 2013), in which parents are prompted, through videos and activities, to reflect on their own behaviour and beliefs, how these might be affecting their children, and identify personal goals for strategy implementation. The prototype version of PWTP comprised two online modules, each taking around 30 min. The prototype was delivered via the online learning platform, edX. The modules contained a series of videos involving narrated voiceover content, video demonstrations of parenting traps and strategies, and video testimonials from parents, children and officials sharing their experiences. The National Rugby League (NRL) and QRL gave permission for the research team to film background footage and testimonials at junior rugby league games and events and connected the team with local clubs to identify families willing to be involved in filming of parenting scenarios. Filming and post-production editing was conducted by the teaching technology and innovation team from the researchers’ university. Each video was followed by a short activity to identify personal areas of concern in sports parenting, reinforce key messages about program content and strategies, and to set goals for putting new strategies into place.

Module 1 covered topics including the importance of sport for children, the role of parents in sport and common sporting problems experienced by parents and children. Through voiceover narration and interactive activities, parents were prompted to identify patterns in their own behaviour and expectations that may be negatively affecting their child’s participation in rugby league. Module 2 provided parents with tips and practical strategies that parents could use to help their child enjoy playing rugby league, including how to give helpful feedback, have a positive and respectful attitude to the game and be a good sport (see Supplementary Table 1).

#### Data Analysis

The interviews were transcribed by a research assistant under the supervision of two of authors SR and CM. Subsequently, the qualitative dataset was abductively analysed following Braun and Clarke’s (2019) guidelines for codebook thematic analysis. The fourth author engaged in the process of familiarisation by immersing himself in the qualitative data. This involved recursively engaging with the transcripts and audio recordings. Next, he inductively identified patterns across the qualitative extracts and assigned labels to create codes across the extracts. These codes were subsequently clustered into higher-level patterns, resulting in the creation of provisional themes. These provisional themes were developed deductively in consideration of the research questions and existing literature. Lastly, through an iterative process of refining and naming themes, together the authors CM and JL determined a final list of themes and subthemes. Parent responses on the CSQ were analysed quantitatively by calculating frequency of scores on each item and an overall mean total score.

## Results

Two themes and six subthemes were generated from the thematic analysis. The first theme involved participants discussing the practicalities of participation in the program, with subthemes related to program endorsement, program refinements, and program barriers. The second theme included the program’s impact on parents, with subthemes related to new and reinforced knowledge, behavioural intentions, and program applicability beyond junior rugby league.

### The Practicalities of Participation

Parents’ perceptions regarding the practicalities of the program were represented across three subthemes: (1) program endorsements, (2) considerations for program refinements, and (3) participation barriers and hesitations. First, the participants *provided endorsements for the program*, indicating that the information covered was important for all individuals in attendance at youth sporting events. Parents appreciated the content and the importance of disseminating this information to parents. Participants liked the brevity and interactivity of the program. Parent 19 stated:

I liked the interactive nature of [the program], particularly the videos. And I like the fact that it didn’t take too long to go through it and that you got feedback straightaway on your answers. And I like the fact that it was local sports. I could see myself in those spaces and recognise how my children act and see those same events and things like that in my own community.

In support of the perceived value of the program, participants emphasised the importance of having all new parents take part. Other parents suggested that the program should be offered to all parents irrespective of their level of experience in rugby league. Parent 4 reported:

I think it’s probably a good idea that a new parent coming to rugby league sit down and do [the program] before they start. Whatever sport that their child is going to participate in, all parents should have to do that, especially if they are new parents to sport, because that shows them how to react and how to encourage a child and how to act on the field…

Second, parents offered *considerations for program refinements*. All participants provided their opinions about the scheduling of the program. Most participants felt the timing of the program could be improved, suggesting that the program should take place before the season or early in the season rather than mid-season. They felt this would better allow parents the opportunity to learn about positive parenting behaviours early on:

I think before the season starts or at sign up they should be given something to do before the children start playing sports because then we know that before the season starts, before any incident happens throughout the season, then we’ve all got a level playing ground and we’ve all got an insight into what is positive and what is good behaviour of the parent. (Parent 6)

There were some contradictory suggestions, with some participants suggesting the program could be shorter, while others suggesting the program could have more depth. Alternative methods of implementation were proposed by some parents, such as group delivery and/or providing a specific time and location for participants to complete the program (e.g., during a training session), whereas others appreciated the independent, self-paced nature of the program.

Third, participants offered *possible barriers and hesitations* of participation that might limit parent involvement in the program. Participants indicated that parents’ busy schedules and time constraints might cause some parents to not participate unless it was mandated. For instance, Parent 10 explained: “Parents feel like they’re busy enough as it is with work and school. To be able to make that time to take your child to training is a big enough ask as it is.”

Another barrier commonly reported by participants was that some parents may feel the information is not applicable to them. For instance, when asked about possible barriers, Parent 19 offered the following: “Some people think that they’re perfect parents, and don’t need to do any further training. I don’t agree with that.” Several participants predicted that the parents that need it the most are the parents that will be least likely to participate, reflecting a lack of open-mindedness to change. For instance: “It’s a struggle to get to get people to look at their own behaviours in a way that they think they need to change, I think people can be pretty naive and don’t see themselves as the ones that are doing those behaviours.” (Parent 15).

### Program Impact

Parents discussed the various ways the program had an impact on them across three subthemes: (4) new and reinforced knowledge, (5) behavioural intentions, and (6) applicability beyond junior rugby league.

Fourth, parents talked about the *new and reinforced knowledge* they gained from the program. Sometimes the knowledge and ideas were new or challenged prior thinking, whereas other content reinforced existing behaviours and/or served as an important refresher. For instance, one parent described how the program reinforced knowledge around positive communication with their child:

The program was a good reminder to stay positive, especially with junior rugby league. I think the course really reinforces to look at the positives, look at what your child does well, focus in on those, and talk to them about how they might be able to improve other skills. (Parent 7)

Another important learning identified by participants was the importance of modelling appropriate behaviours, such as emotional regulation and being respectful towards coaches and officials, and how this contributed to a more positive environment for the children. For instance, when asked to provide some of the takeaway messages from the program, Parent 9 described:

You’ve also got to be your child’s number one role model. And I think that fundamentally your behaviour is the thing that’s probably going to be the most important thing that really shapes your child’s thoughts on their sport to a degree.

Fifth, when asked about their *behavioural intentions* to try out the strategies in the program, some participants described that they had already applied the knowledge learned. Most participants explained that they had not yet started to apply the strategies in the program but emphasised their intentions to engage in behaviours in the future that aligned with the knowledge they gained, such as providing positive encouragement and constructive feedback, regulating their own emotions, having a greater focus on enjoyment, and letting their child approach them before providing feedback. Parent 16 explained:

I would definitely try to get more involved with volunteering and be the best supporter I can be. And that’s all that the kids want. Showing positive reinforcement that we are there to watch him play the game and we know that he’s doing his best.

Lastly, parents highlighted that the knowledge and strategies learned through the program were *applicable beyond junior rugby league*. They described how they thought the content of PWTP was applicable to any sport at any level. For instance:

I think the program is terrific… I’d love to see it rolled out across all junior rugby league clubs, so that they can make it available to their parents. I think, you know, ultimately, you’d be doing it for netball and basketball and all the other junior sports too. (Parent 9)

Parents also strongly felt that the content of PWTP could be generalised to contexts outside of sport, particularly the home environment. This was well exemplified by Parent 9 who described:

The tips were helpful, yes, not necessarily just in terms of how I engage with my child directly associated with NRL but at home even. One of the things I did like … was about picking and choosing the time to actually talk to my son about his game. I felt that that might actually translate at home, to when mistakes are made. So, I’ve actually taken that away as a really big thing to let that temperature cool and let him come to me when he is ready to talk.

### Program Satisfaction

**Fig. 1:**
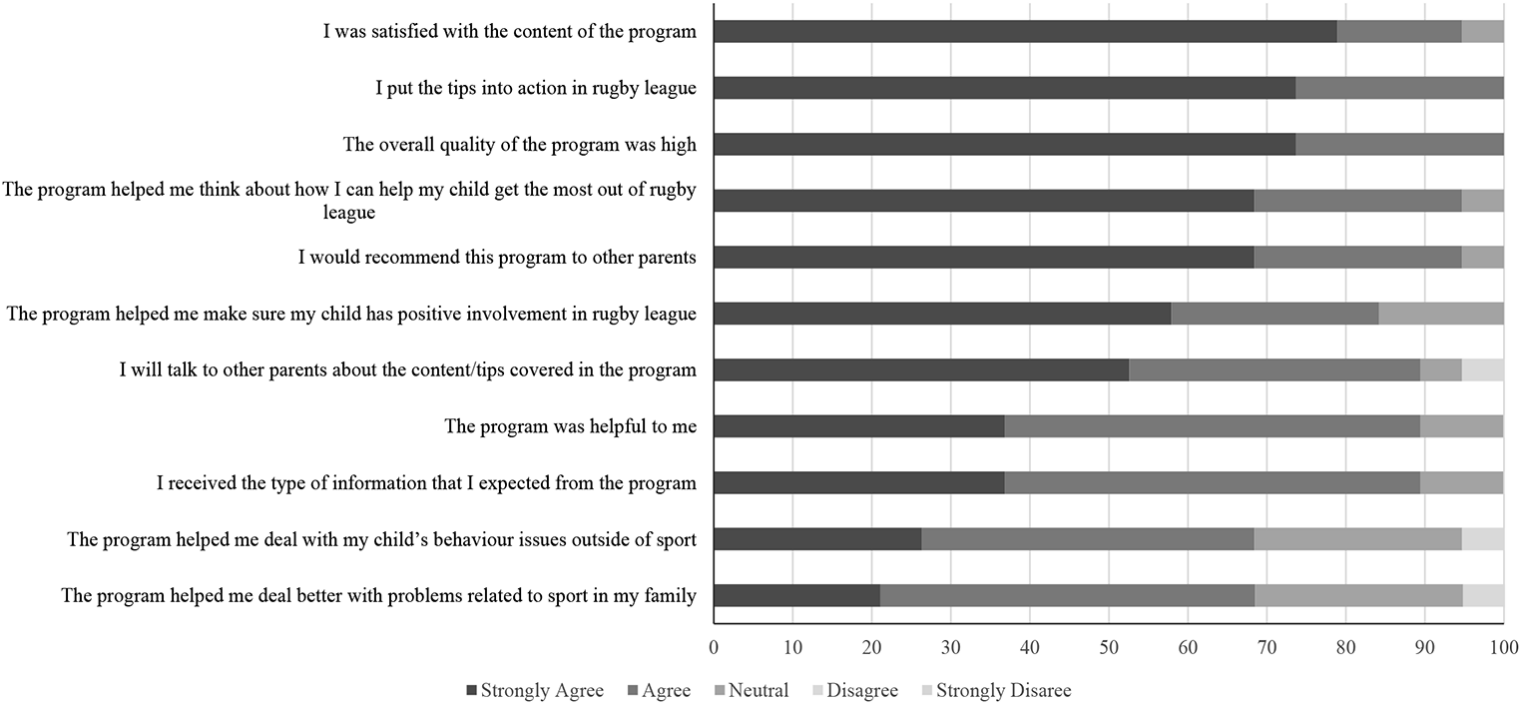
Parent ratings of satisfaction with play well triple P in Study 1 (*N* = 19)

The frequency of parents’ ratings on each item of the CSQ is displayed in Fig. 1. Endorsement of the program (based on the proportion of parents who responded agreed or strongly agreed) was highest for the content of the program, overall quality of the program, the relevance of the program (i.e., putting the tips into action, helping parents think about how they can help their child get the most out of rugby league, and the program helping parents make sure their child has positive involvement in rugby league). Notably, approximately 95% of parents agreed that they would recommend the program to other parents. Parents had the lowest endorsement for items related to the generalisability of the program content to whole family and to their child’s behaviour outside of sport.

The mean score for all 11 items was 4.42 (*SD* = 0.35).

## Summary of Results

The results from the interviews and ratings on the CSQ suggested that parents were generally satisfied with the prototype of Play Well Triple P and endorsed the focus of the program on parenting behaviour in sport. Findings from the interviews and CSQ supported the potential impact of the program, particularly in terms of its influence on parents understanding their role in helping their children have a positive experience in rugby league. Strengths of the program highlighted by parents included its applicability and relevance to participants as sporting parents, the interactive delivery format, and the usefulness of the content and strategies presented in the program. Although items related to the generalisability of the program had the lowest satisfaction ratings on the CSQ, during the interviews parents highlighted the relevance of the program to all adults involved in junior rugby league, and to their children’s involvement in other contexts outside of rugby league.

Parent feedback also provided insight into barriers that may prevent parents from completing the online modules and/or implementing the strategies with their child. This included feedback regarding optimal timing for delivery, challenges of fitting the program into busy family schedules, difficulties finding time to put the strategies into practice, and helping parents understand the relevance of such a program. There was mixed feedback on the length and intensity of the program, with some parents appreciative of the program’s brevity and others commenting that the program could have had more depth. Overall, however, the feedback suggested that it was important that the program required only a small time commitment from parents, and that there needed to be a mechanism for encouraging parental implementation of strategies throughout the season. This feedback, in combination with the need to maximise the reach of the program to as many parents as possible, led the research team to consider several refinements to program length and delivery format. Further, while parents were satisfied the content of the prototype, parents were concerned that not all parents would perceive the program as relevant or engaging. As such, the research team also sought to find ways that would optimise engagement from potentially resistant parents. These refinements are described in Study 2.

## Study 2

### Aims and Hypotheses

There were three major aims for Study 2. First, the aim was to use the feedback obtained from Study 1 to refine the program and develop a pilot version of PWTP. These refinements and how they were achieved are described at the outset of the Method section. The second aim of the study was to assess implementation feasibility of PWTP in terms of uptake of the program, and the level of satisfaction and acceptability with the program. The third aim of Study 2 was to quantitively evaluate the effectiveness of PWTP for those parents who took part in the program. Aims 2 and 3 were addressed by trialling the new version of PWTP with a separate and larger sample of parents of junior rugby league players (aged 5 to 12 years) in a non-randomised feasibility study (Eldridge et al. 2016). Of specific interest were the effects of PWTP on sports-related and general parenting practices, parental expectations for their child’s sport, and child behaviour from pre- to post-intervention. It was hypothesised that, from pre- to post-intervention, participation in PWTP would be associated with (a) reductions in negative and increases in positive sports parenting behaviour; (b) reductions in unhelpful and increases in helpful parental expectations about sport; (c) reductions in ineffective parenting practices in the home; and (d) reductions in child behaviour problems.

### Method

#### Refinement of Prototype Version of Play Well Triple P

Two major changes were made to PWTP in this phase: (1) condensing online content from two modules to one; and (2) addition of text message prompts.

##### Refinement of Online Modules

Two major changes were made to the online module: (1) reducing the two online modules in the prototype version down to one module; and (2) incorporating strategies to promote engagement in the program. Based on the feedback obtained in Study 1 related to barriers to engagement, consultation with our research partners at the NRL and QRL, and an external review of the module content by developers of other Triple P programs, it was decided that to support parent completion, the online component needed to be distilled into one module. This needed to be done in a way that maintained the key knowledge, messages and strategies embedded in the two-module version of the program. The researchers consulted with a professional film production company to condense the video footage, and to identify strategies for maximising engagement in the videos being used in the online module. Working alongside the production company, the script was revised, the footage and voiceover content was streamlined, and the content was better organised in a way that ensured program messaging was clear, engaging and regularly reinforced during the video footage. In addition, to support acceptability of the program, the narration content was re-recorded using a major rugby league stadium in Brisbane, Queensland as the backdrop, and a well-known ex-rugby league player volunteered his time to be filmed as the ‘host’ of the program. The host along with authors CD and MS, who are registered psychologists, delivered the narration for the videos.

The content was conceptualised into four video segments, which ranged in length from 2:00 min to 6:40 min. Video content totalled just over 19:00 min. The segments were embedded within the Triple P Online (Turner and Sanders 2011) bespoke internet platform with six interactive activities designed to promote self-regulatory behaviour related to sports parenting (see Supplementary Table 1 for an overview of program content). The entire module, including watching all four videos and engaging in each activity, was designed to take no longer than 40 min.

##### Addition of Text Message Component

A text message component was added to the program in response to parent feedback in Study 1 regarding behavioural intentions to implement strategies. The addition of the text messages preserved the brevity of the program, while aiming to: (a) reinforce key program messages throughout the season; (b) prompt parents to implement the strategies and act on personal goals from the online module; (c) facilitate the maintenance of any short-term positive effects of the online module over the course of the season. This decision was consistent with strong evidence for the efficacy of text message interventions for promoting behaviour change (Armanasco et al. 2017; Head et al. 2013), as well as prior evidence that incorporating text messages into evidence-based parenting programs can facilitate strategy implementation and program engagement (Bigelow et al. 2020; Murray et al. 2015).

Text messages were scheduled for delivery upon completion of online module using the propelo™ text message management platform on a biweekly schedule. The first message each week was delivered in the lead-up to the game (e.g., at weekly training, or on a Thursday before a weekend game) and the second was delivered after the game. The message framework contained 12 pairs of lead-up and game-day messages. The lead-up messages prompted parents to try out strategies in upcoming games (e.g., “When we focus on winning, too much pressure is placed on children. Can you think of something positive to focus on in {child name}’s game this week?”). The game day messages prompted parents to reflect on their behaviour (including efforts to try out strategies) and their child’s reactions (e.g., “Talk with {child name} about the skill they wanted to practice today BUT choose your time wisely. On the way home may not be the best time!”).

Messages were tailored to the parent (i.e., by addressing them by name, referring specifically to their child’s name), and incorporated responses entered in the online module related to the specific goals the parent had set for their behaviour. To promote engagement, four of the messages (two lead-up messages, two game-day messages) prompted parents to text back with a yes or no response, with a pre-determined response sent back to parents depending on their answer. Two follow up messages were sent to parents at the conclusion of the study to access further parenting support if needed and to thank them for their participation in the program.

#### Participants and Recruitment for Evaluation of Play Well Triple P

Participants were parents or caregivers of children aged 5 to 12 years enrolled in junior rugby league in the 2021 season. Consistent with recommendations provided by parents in Study 1 regarding the timing of the intervention, recruitment for the study began at the start of the 2021 junior rugby league season (i.e., across April and May) to enable parents to complete the online pre-intervention assessment (see below) and enrol in the online module as early as possible. Parents were recruited via outreach through rugby league social media channels, traditional media and emails sent from the NRL to parents registered with Southeast Queensland junior rugby league clubs. Promotion materials invited parents to register their interest in the study via the project website. Details regarding the demographic characteristics of the final sample of parents and target children is provided at the outset of the Results.

#### Evaluation Measures

Evaluation measures were a series of validated and psychometrically tested questionnaires, which were embedded in one online survey. Demographic items were completed at pre-intervention, while the CSQ was completed at post-intervention. All other measures were completed at both time points, and all measures were completed online. Where parents had more than one child in the age range enrolled in rugby league, parents nominated a target child to focus on when completing the program and the evaluation measures.

##### Demographic Information

Parents provided demographic information regarding their own and their child’s age and gender. Parents were also asked to provide their highest level of education, employment status, household income, ethnic and cultural background, relationship status and number of children. Parents also responded to questions about the family’s involvement in rugby league, including how many seasons the child had played rugby league, how many other children in the family play rugby league, and whether the parent volunteers (and in what capacity) at the child’s club.

##### Sports Parenting Behaviour

The 20-item Parental Involvement in Sport Questionnaire (PISQ; Lee and MacLean 1997) was used to assess negative and positive parenting behaviours that occur in the context of junior sport. The Directive Behaviour subscale (α = 0.89^1^) was used to measure negative sports parenting and assessed the extent to which parents engaged in controlling, intrusive or pressuring behaviour towards their child in sport (e.g., “after a game, competition, or performance, I tell my child what I think they did badly”). The Active Involvement and Praise and Understanding subscales were used to assess positive sports parenting behaviour. The Active Involvement subscale (α = 0.73) assessed the extent to which parents were active in their child’s sport (e.g., “I volunteer to help out at games”), while the Praise and Understanding subscale (α = 0.79) assessed levels of praise and empathy parents displayed towards their child (e.g., “after a game, competition or performance, I praise my child regardless of whether they won or lost”). All items were rated on a 5-point scale from 1 (*never*) to 5 (*always*). Higher scores indicated greater use of the parenting approach.

##### Parental Beliefs and Expectations About Sport

Two subscales from the Perception of Success Questionnaire (POSQ; Roberts et al. 1998) were used to assess parental expectations towards their child’s involvement in sport. The POSQ was developed to assess the extent to which individuals endorse either task-oriented or ego-oriented goals for participation in sport. The items were reworded for this study for parent-report to assess a parent’s goals for their child’s participation in sport. The 8-item Task Orientation subscale (α = 0.71) measured parental goals related to skill development and personal improvement (e.g., “when my child plays sport, I feel they are most successful when they perform to the best of their ability”). In comparison, the 8-item Ego Orientation subscale (α = 0.78) assessed parental goals focused on the child winning or being the best (e.g., “when my child plays sport, I feel they are most successful when they show other people they are the best”). All items were rated on a 5-point Likert scale from 1 (*strongly disagree*) to 5 (*strongly agree*). Total scores were created for each subscale, with higher scores reflecting greater valuing of that goal for their child.

Parental competitiveness was measured using the 12-item General Competitiveness subscale (α = 0.94) from the Competitive Orientation Measure (Newby and Klein 2014). Parents were asked to report their personal levels of competitiveness in sport (e.g., “I get a lot of enjoyment from competition”) on a 5-point Likert scale from 1 (*strongly disagree*) to 5 (*strongly agree*). Higher scores reflected greater competitiveness.

##### Parenting Practices in the Home

The short form of the Parenting Scale (Reitman et al. 2001) was used to assess participants’ use of ineffective parenting practices in the home. Each item was rated on a 7-point Likert scale, with a less appropriate and more appropriate anchor for each item. Two subscales from this questionnaire were used. The Laxness subscale (α = 0.85) assessed inconsistent and permissive parenting (e.g., “If my child gets upset when I say ‘No’ … I back down and give in. vs. I stick to what I said”), while the Over-reactivity subscale (α = 0.78) assessed hostile and coercive verbal and emotional reactions when parenting (“When my child misbehaves… I raise my voice or yell vs. I speak to my child calmly”). Total scores were calculated for each subscale, with higher scores indicating higher levels of ineffective parenting.

##### Child Behaviour Problems

Parents’ perception of their child’s behaviour was measured using the Conduct Problems and Hyperactivity subscales of the Strengths and Difficulties Questionnaire (SDQ; Goodman 1997). The Conduct Problems subscale (α = 0.69) comprised 5 items assessing children’s defiant or aggressive behaviour (e.g., “Often fights with other children or bullies them”), while the Hyperactivity subscale (α = 0.83) assessed difficulty with concentration and over-activity (e.g., “Constantly fidgeting or squirming”). Parents rated each item using a 3-point Likert scale from 0 (*not true*) to 2 (*certainly true*), with higher scores indicating more behaviour problems.

##### Program Satisfaction

Parents completed a similar version of the Client Satisfaction Questionnaire (CSQ) that was used in Study 1. The Study 2 version involved 12 statements about the participants’ experience with the program, all rated on a 5-point scale from 1 (*Strongly Disagree*) to 5 (*Strongly Agree*), with higher ratings reflecting greater satisfaction. Nine items were the same as those used in Study 1. One item, “The program has helped me to deal more effectively with problems that arise related to sport in my family” was replaced with a more straightforward item, “I used the tips I learned in Play Well in other sports my children play”, while the item, “The program has helped me think about the things that I can do to help my child get the most out of playing rugby league” was removed. Two additional items were added to assess satisfaction with the text message component of PWTP; “I found the text messages helpful” and “The number of text messages that I received was appropriate”. Finally, parents were asked if PWTP prompted them to seek further parenting support.

#### Procedure

The study was approved by the authors’ institutional human research ethics committee (approval #: 2,020,001,179). All parents gave informed consent to all aspects of program completion and data collection before participating. All recruitment materials (e.g., social media posts, emails to parents from the NRL) directed parents to register their interest in the study via a dedicated study website. Parents were then contacted by email to provide information about the study and to direct them to complete the pre-intervention survey, which contained all the measures described above. Each parent was sent a personalised survey link that allowed them to complete the informed consent process and pre-intervention survey via the online survey platform, Qualtrics™. Parents were asked to complete the first survey within 5 days and received up to four weekly email or text message reminders.

Upon completion of the pre-intervention (T1) survey, participants were registered within the PWTP management system, which sent an email to parents with a unique activation code and instructions on how to register in the online component of PWTP. Parents were asked to complete the online module within one week. Parent registrations and module completion was tracked via the PWTP management system, and parents were given up to four email or text message reminders to complete the program. Once parents completed the online module, the text message component of the intervention was activated via propelo™. Parents were sent a text message to their nominated mobile phone number, which they were asked to respond to if they wished to activate the text message component of PWTP. In the last week of the junior rugby league season, parents were emailed a personalised link to complete their post-intervention (T2) survey online and were sent reminders to complete the survey on the same schedule as before. Each survey took parents approximately 20–25 min to complete.

#### Data Analysis Approach

Independent groups t-tests and Chi square analyses on all sociodemographic and baseline variables checked for any systematic biases in participant attrition. Repeated measures multivariate analyses of variance (MANOVAs) were conducted on sets of related dependent variables: sports-parenting behaviour (PISQ Directive Behaviour, Active Involvement and Praise and Understanding subscales); parental expectations about sport (POSQ Task Orientation and Ego Orientation subscales); parenting practices in the home (PS Over-reactivity and Laxness subscales); and child behaviour (SDQ Conduct and Hyperactivity subscales). Where multivariate effects were found, univariate *F* values were examined to determine which variables contributed to the multivariate effect. A paired samples *t*-test was conducted on the individual parental competitiveness variable. Levels of clinically meaningful change from pre- to post-intervention was evaluated using effect sizes (Cohen’s *d*).

## Results

### Data Preparation

Missing values analysis was conducted on all study variables separately for pre-intervention and post-intervention data. The results of Little’s MCAR test (Little 1988) indicated that the pre-intervention data was not missing completely at random, *Χ*^2^ (837) = 970.85, *p* < .001, while the post-intervention data was missing completely at random *Χ*^2^ (648) = 680.58, *p* = .182. However, there was very minimal item-level missing data within each questionnaire measure, with most measures having no missing data, and < 1.0% missing data on the SDQ and PERS. Thus, the Estimation Maximisation (EM) method was used to impute missing data within each questionnaire at both time points.

### Participant Flow and Intervention Completion

In total, 414 parents registered their interest via the study website. These parents were invited to participate in the study and were sent a unique link to access the informed consent process and the T1 survey. From this group, 261 parents gave informed consent, completed the T1 survey and were sent a personalised code to access the PWTP online module. Of these parents, 221 registered themselves in the online platform (84.6% of T1 completers), with 144 parents completing the online module (55.1% of T1 completers). Parents who completed the online module were then sent a text message via the propelo™ system to activate the text message component of the intervention. Twenty-five parents did not receive text messages because they did not respond to the activation text message confirming that they wished to proceed (*n* = 22 parents) or actively opted out of the text messages (*n* = 3). Thus, 120 parents (46.0% of T1 completers) received both the online module and text message components of PWTP. In comparison, 101 parents (38.7% of T1 completers) completed at least the online module and had T1 and T2 data available.

A series of independent groups t-tests for continuous variables and chi square analyses for categorical variables were conducted to compare sociodemographic characteristics and T1 scores on the questionnaire measures of parents who completed T1 and the PWTP online module (completers, *n* = 144) versus those who completed T1 but did not complete the online module (non-completers, *n* = 117). In terms of sociodemographic background, there were no significant differences between completers and non-completers in parent or child age, parent gender, educational attainment, employment status, couple relationship status, identified cultural background, or household income. The only significant difference was for target child gender, with a smaller proportion of female junior rugby league players among parents who completed the PWTP online module (3.5%) compared to those who did not (14.5%), *Χ*^2^ (261) = 10.23, *p* < .001.

There were also no significant differences between completers and non-completers in T1 scores across the evaluation measures. Specifically, there were no differences between completers and non-completers in sports parenting behaviour (PISQ Directive Behaviour, Praise and Understanding, Active Involvement), parental expectations about children’s sport (POSQ Ego Orientation, Task Orientation), parental competitiveness, parenting practices in the home (PS Over-reactivity, Laxness), or child behaviour (SDQ Conduct Problems, Hyperactivity).

### Characteristics of Parents Who Completed Play Well Triple P

As this study was a quasi-experimental feasibility study and did not include a control group, a per protocol approach to analyses was employed. All participants who completed at least the online module and had T1 and T2 data available were included in analyses (*n* = 101 parents). The final sample of parents ranged in age from 25 to 56 years (mean age = 38.42 years; *SD* = 6.06), with both mothers (72.4%) and fathers (27.6%) taking part in the evaluation. While most of the sample identified as being Australian (78.1%), there was representation from Indigenous Australian (8.6%), New Zealand Pākeha (2.9%), New Zealand Māori (1.9%) and Polynesian (3.8%) cultural backgrounds. Most parents were in paid full-time or part-time employment (83.8%), with annual household incomes ranging from $20,000 to over $200,000. Approximately 41.0% of parents reported an annual household income below the median Australian household income in 2020^2^. In terms of highest level of education, 37.2% of parents had completed an undergraduate or postgraduate university degree, 29.5% had a vocational qualification, and 21.9% had completed secondary school. The remaining 11.4% had less than a secondary school education. Most parents were married or in cohabiting relationships (82.0%), with 13.3% identifying as single parents, and 5.7% as separated or divorced. Finally, three quarters of the sample (75.2%) volunteered at their child’s club, including 18.1% who were coaches. Target children ranged in age from 5 to 12 years (mean age = 8.57 years; *SD* = 1.89), and most were male (96.2%). This was the first junior rugby league season for 26.7% of the target children, with relatively equal proportions participating in their second to their fifth seasons.

### Post-intervention Outcomes

Time 1 and 2 descriptive statistics and the results of outcome analyses are presented in Table 1. A repeated measures MANOVA on the three domains of sports parenting behaviour (PISQ) revealed a significant multivariate effect of time, *F*(3, 98) = 17.22, *p* < .001. Examination of the univariate effects indicated that there was a significant decrease in Directive Behaviour (moderate effect) and an increase in Active Involvement (small effect) from T1 to T2 among parents who completed at least the PWTP online module. In comparison, there was no multivariate effect of time for parental expectations about sport (POSQ; *F*(2, 99) = 0.29, *p* = .751), parenting practices in the home (PS; *F*(2, 93) = 1.67, *p* = .193) or child behaviour (SDQ; *F*(2, 93) = 1.19, *p* = .309). At the univariate level, the effect of time for parental over-reactivity approached significance (*p* = .070). Finally, a paired samples *t*-test indicated that there was no significant change in parents’ level of general competitiveness from T1 to T2, *t*(93) = 1.29, *p* = .202.

**Table 1:** Descriptive statistics and results of outcome analyses for participating parents

	T1 mean (SD)	T2 mean (SD)	Univariate F value	*p*	Cohen’s d
PISQ Directive Behaviour	21.83 (7.76)	18.63 (6.17)	29.20	< 0.001	0.46
PISQ Praise & Understanding	22.53 (2.33)	22.32 (2.53)	0.66	0.420	0.09
PISQ Active Involvement	9.43 (3.50)	10.35 (3.47)	14.01	< 0.001	-0.26
POSQ Ego Orientation	15.17 (5.50)	14.77 (5.61)	0.55	0.459	0.07
POSQ Task Orientation	28.26 (2.03)	28.24 (2.25)	0.01	0.928	0.01
General Competitiveness	8.52 (10.70)	7.62 (10.65)	1.29^a^	0.202	0.09
PS Over-reactivity	3.00 (0.97)	2.85 (1.01)	3.36	0.070	0.15
PS Laxness	2.23 (0.89)	2.21 (0.91)	0.07	0.795	0.02
SDQ Conduct	1.87 (1.95)	1.96 (2.03)	0.32	0.570	-0.05
SDQ Hyperactivity	4.38 (2.65)	4.13 (2.75)	1.53	0.219	0.09

Note PISQ = Parental Involvement in Sport Questionnaire; POSQ = Perception of Success Questionnaire; PS = Parenting Scale; SDQ = Strengths and Difficulties Questionnaire

^a^ Paired samples *t* statistic

### Program Satisfaction

**Fig. 2:**
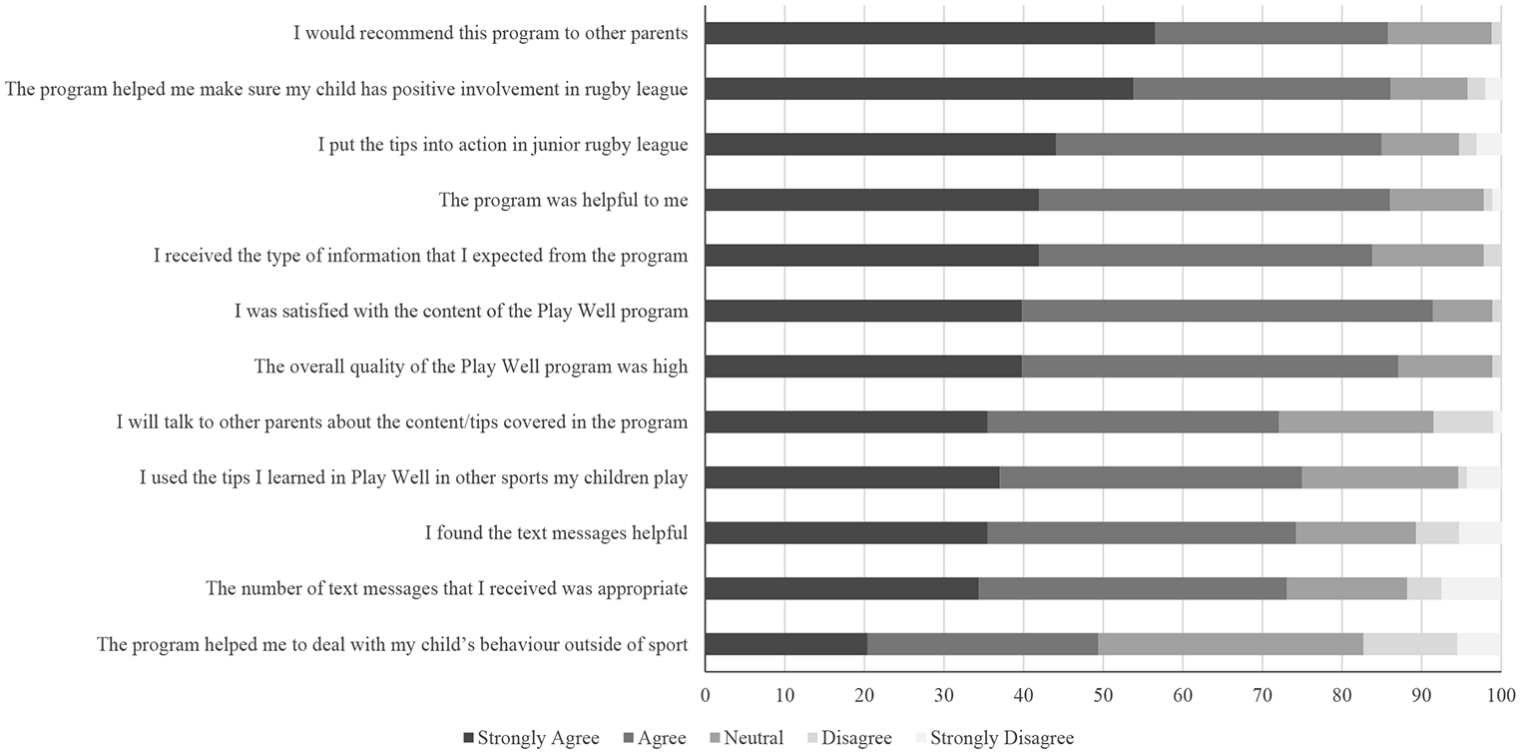
Parent ratings of satisfaction with play well triple P in Study 2 (*N* = 93)

The CSQ was completed by 93 parents (92.1% of the final sample). In terms of the proportion of parents who strongly agreed or agreed with each statement, aspects of the program that had the highest level of endorsement included the helpfulness of the program (generally and specifically in supporting their child’s positive involvement in rugby league), the content and information covered by the program, and the overall quality of the program (see Fig. 2). Like Study 1, there was lower endorsement from parents for statements related to the generalisability of the program (i.e., applying tips to other sports their child plays; helping parents deal with behaviour outside of sport).

Further, parent satisfaction was lower for the text messages in comparison to other elements of the program. However, still over 70.0% of parents endorsed items on the CSQ related to the text messages. Importantly, 85.8% of parents indicated they would recommend PWTP to other parents. 14% of parents (*n* = 13) who completed the CSQ indicated that they had sought further support for their parenting after participating in PWTP. The mean score for all 12 items was 4.10 (*SD* = 0.64), which was slightly lower than the mean in Study 1 (4.42), but consistent with satisfaction scores for other online and self-directed variants of Triple P (e.g., Sanders et al., 2012, 2014a).

## General Discussion

The findings from Studies 1 and 2 provided important information regarding the potential effects of the program on sports parenting as well as parental perceptions of the usefulness and feasibility of the program. The main finding from Study 2 was that participation in PWTP was associated with improved sports parenting behaviour. Consistent with hypotheses, parents who completed PWTP reported increased positive involvement and decreased negative sports parenting behaviour from pre- to post-intervention. In contrast, participation in PWTP did not influence parental expectations about children’s involvement in sport, personal levels of competitive attitudes, nor child behaviour. Nevertheless, this study supported the positive findings from prior evaluations of sports parenting interventions including those conducted face-to-face (e.g., Thrower et al. 2017 for junior tennis; Dorsch et al. 2017 for junior soccer) and one adapted for online delivery (Thrower et al. 2019 for junior tennis). The current study extended this work into the Australian context of junior rugby league and showed that a low-intensity, self-directed program can improve sports parenting.

This study, along with prior research evaluating the usefulness and effectiveness of other sports parenting programs (e.g., Thrower et al. 2019), indicated that providing parents with positive and constructive strategies for supporting their children in sport produces important improvements in sports parenting behaviour. Several studies have demonstrated that parental pressure and directive and controlling parenting practices are related to negative child sporting outcomes, such as lower enjoyment and motivation (Amado et al. 2015; Sánchez-Miguel et al. 2013), competition anxiety (Bois et al. 2009; O’Rourke et al. 2011), and sport dropout (Crane and Temple 2015). Thus, programs that make meaningful improvements to parental behaviour should have corresponding positive effects on children’s participation in and enjoyment of sport. Further research involving controlled trials that evaluate child sports outcomes are needed to properly test the benefits of sports parenting programs.

One notable additional finding from Study 2 was that participation in PWTP was associated with a trend (*p* = .07) towards reducing over-reactive parenting practices at home. While not significant, likely due to the small sample size and limited power to detect small effects, this finding suggested that parents may have applied the positive parenting strategies described in PWTP from the sports to the home context. Accessibility to services along with the stigma attached to seeking support for parenting are often identified as key barriers to parental engagement in evidence-based parenting programs (Mytton et al. 2014). Junior sport, therefore, may present a non-stigmatising, acceptable, and readily accessible context for the delivery of evidence-based parenting strategies, as well as an entry point for parents seeking further support for parenting or child behaviour problems. Indeed, 14% (*n* = 13) of parents who completed the Client Satisfaction Questionnaire at the end of the rugby league season indicated that involvement in PWTP prompted them to access additional parenting support.

Overall, parents indicated that they were highly satisfied with PWTP, including the quality and content of the program, the usefulness of the online and text message components, and the relevance and usefulness of the content. While satisfaction was high for those parents who completed the program, it is important to note that there was significant drop-out at each stage of the research. Despite strong initial interest from parents in the program, with 414 registrations on the project website, only 120 parents completed the online module and were sent the text message framework. Other research with online parenting programs has documented that maintaining engagement of parents throughout the course of a program is a significant challenge (Day et al. 2021). In this study, attrition of parents seemed to be related both to the demands of the research (e.g., 37% [*n* = 153] of parents who initially registered their interest did not go on to complete the T1 assessment), as well as engagement in the program itself. For instance, 15% [*n* = 40] of the 261 parents who completed the T1 assessment did not activate their registration in the PWTP online module, while 35% [*n* = 77] of the 221 parents who registered in the online module did not complete it. The attrition at each transition point in the pilot trial has implications for implementation, pointing to the need to avoid multiple steps in the registration process and ensure parents enter the online module and have the text message component activated in as streamlined and automated a manner as possible. Many junior sporting organisations now incorporate team management apps to manage scheduling and communication with players and families. These apps could be leveraged to deliver PWTP video and messaging content in a seamless and user-friendly way for families. Parental engagement is also likely to be enhanced if PWTP sits within a larger communications strategy in a club or organisation that reinforces messaging around positive parenting in sport.

Along with challenges related to retention in Study 2, other characteristics of the sample should be considered when interpreting the findings of this research. Consistent with the player profile of junior rugby league players (which is open to children aged 5 to 12 years), most target children were male (96.2%) and were on average around 8.5 years old. The available research on sports parenting programs (Dorsch et al. 2017; Thrower et al. 2017) has targeted a comparable age range of children, consistent with an early intervention approach to preventing challenging parental behaviour that has been reported in research with adolescents (e.g., Elliott and Drummond 2017a, b; Knight et al. 2011; Tamminen et al. 2017). Research is needed to examine whether sports parenting programs are effective with parents of adolescents and what, if any, modifications might need to be made to program content or delivery to ensure program efficacy. Further, research should examine the application of programs like PWTP to other sports, particularly individual sports or those involving female players.

Another potential limitation in the present sample was that it was predominantly mothers (72.4%). While it may seem low that only 27.6% of the sample was fathers, this rate was somewhat higher than is often seen in parenting intervention research. For comparison, participation of fathers in trials of other online variants of Triple P was reported at lower than 10% (e.g., Baker et al. 2017; Day and Sanders 2018; Sanders et al. 2012). Father engagement has long been a challenge in parenting intervention research, with participation rates from fathers typically well below that of mothers (Gonzalez et al. 2023). The slightly higher participation rate of fathers in this study may reflect research findings that sport is a familiar and comfortable cultural context for father involvement with their children (Coakley 2006; Harrington 2006). While much more research is needed, it may be that junior sport offers an important engagement avenue for fathers looking to gain support and strategies to improve their parenting.

Another important limitation was that this study was a quasi-experimental feasibility study with no randomised control condition, which limits the ability to derive causal inferences about the effects of the program. Further, a longer-term follow-up assessment was not conducted, so it is not known whether the short-term intervention effects on sports parenting behaviour were maintained into the next sporting season. Finally, outcomes were assessed using self-report parent questionnaires only. Thus, future research should employ a randomised controlled trial research design, assess outcomes over the longer-term, and use multi-informant assessments.

## Summary

In summary, this multi-stage study used an iterative approach to program design and development to develop a unique blended modality program. Findings indicated that parents perceived that there was a need for a program focusing on positive parenting in sport and that PWTP was an engaging and high-quality approach to delivering a sports parenting intervention. Further, the findings from Study 2 indicated that parental participation in PWTP is associated with improvements in sports parenting behaviour. Although PWTP was developed as a bespoke intervention for junior rugby league, given the promising findings from the present study, a similar approach could be applied to other team (e.g., soccer, basketball) and individual sports (e.g., tennis, swimming, athletics) wishing to support parents and children to engage positively in sport over time. Ultimately, programs like PWTP should act as an important component of a broader, integrated player development and participation framework to help ensure that children derive the physical, psychological, and social benefits provided by participation in organised sport.

## Electronic Supplementary Material

Below is the link to the electronic supplementary material.

Supplementary Material 1

## Data Availability

The data that support the findings of this study are available from the corresponding author upon reasonable request.

## Footnotes

1 All coefficient alpha values reported in the measures section were based on the current sample.

2 The median household income in Australia in the 2019–2020 financial year was $92,872 (ABS, 2020).

## References

[CR1] Amado D, Sánchez-Oliva D, González-Ponce I, Pulido-González JJ, Sánchez-Miguel PA (2015) Incidence of parental support and pressure on their children’s motivational processes towards sport practice regarding gender. PLOS ONE (Vol 10(6):e0128015. 10.1371/journal.pone.0128015 10.1371/journal.pone.0128015 PMC4454433 26039062

[CR2] Armanasco AA, Miller YD, Fjeldsoe BS, Marshall AL (2017) Preventive health behaviour change text message interventions: a meta-analysis. Am J Prev Med 52(3):391–402. 10.1016/j.amepre.2016.10.042 28073656 10.1016/j.amepre.2016.10.042

[CR5] Baker S, Sanders MR, Turner KMT, Morawska A (2017) A randomized controlled trial evaluating a low-intensity interactive online parenting intervention, triple P online brief, with parents of children with early onset conduct problems. Behav Res Ther 91:78–90. 10.1016/j.brat.2017.01.016 28167330 10.1016/j.brat.2017.01.016

[CR6] Barlow J, Coren E (2018) The effectiveness of parenting programs: a review of Campbell Reviews. Res Social Work Pract 28(1):99–102. 10.1177/1049731517725184

[CR7] Bigelow KM, Walker D, Jia F, Irvin D, Turcotte A (2020) Text messaging as an enhancement to home visiting: building parents’ capacity to improve child language-learning environments. Early Child Res Q 51:416–429. 10.1016/j.ecresq.2019.12.010

[CR8] Bois JE, Lalanne J, Delforge C (2009) The influence of parenting practices and parental presence on children’s and adolescents’ pre-competitive anxiety. J Sports Sci 27(10):995–1005. 10.1080/02640410903062001 19847683 10.1080/02640410903062001

[CR9] Braun V, Clarke V (2019) Reflecting on reflexive thematic analysis. Qualitative Res Sport Exerc Health 11(4):589–597

[CR11] Burke S, Sharp L-A, Woods D, Paradis KF (2021) Enhancing parental support through parent-education programs in youth sport: a systematic review. Int Rev Sport Exerc Psychol 1–28. 10.1080/1750984X.2021.1992793

[CR12] Coakley J (2006) The good father: parental expectations and youth sports. Leisure Stud 25(2):153–163. 10.1080/02614360500467735

[CR13] Crane J, Temple V (2015) A systematic review of dropout from organised sport among children and youth. Eur Phys Educ Rev 21(1):114–131. 10.1177/1356336X14555294

[CR15] Day JJ, Sanders MR (2018) Do parents benefit from help when completing a self-guided parenting program online? A randomized controlled trial comparing Triple P Online with and without telephone support. Behav Ther 49(6):1020–1038. 10.1016/j.beth.2018.03.002 30316482 10.1016/j.beth.2018.03.002

[CR14] Day JJ, Baker S, Dittman CK, Franke N, Hinton S, Love S, Sanders MR, Turner KMT (2021) Predicting positive outcomes and successful completion in an online parenting program for parents of children with disruptive behaviour: an integrated data analysis. Behav Res Ther 146:103951. 10.1016/j.brat.2021.103951 34507006 10.1016/j.brat.2021.103951

[CR16] Dorsch TE, King MQ, Dunn CR, Osai KV, Tulane S (2017) The impact of evidence-based parent education in organised youth sport: a pilot study. J Appl Sport Psychol 29(2):199–214. 10.1080/10413200.2016.1194909

[CR17] Dorsch TE, Wright E, Eckardt VC, Elliott SK, Thrower SN, Knight CJ (2021) A history of parent involvement in organised youth sport: a scoping review. Sport Exerc Perform Psychol 10(4):536–557. 10.1037/spy0000266

[CR18] Dowell TL, Waters AM, Usher W, Farrell LJ, Donovan CL, Modecki KL, Zimmer-Gembeck MJ, Castle M, Hinchey J (2021) Tackling mental health in youth sporting programs: a pilot study of a holistic program. Child Psychiatry Hum Dev 52(1):15–29. 10.1007/s10578-020-00984-9 32246362 10.1007/s10578-020-00984-9

[CR20] Eime RM, Young JA, Harvey JT, Charity MJ, Payne WR (2013) A systematic review of the psychological and social benefits of participation in sport for children and adolescents: informing development of a conceptual model of health through sport. Int J Behav Nutr Phys Activity 10:98. 10.1186/1479-5868-10-98 10.1186/1479-5868-10-98 PMC3751802 23945179

[CR19] Eime RM, Harvey JT, Charity MJ, Casey MM, Westerbeek H, Payne WR (2016) Age profiles of sport participants. BMC Sports Sci Med Rehabilitation 8:6. 10.1186/s13102-016-0031-3 10.1186/s13102-016-0031-3 PMC4788892 26973792

[CR21] Eldridge SM, Lancaster GA, Campbell MJ, Thabane L, Hopewell S, Coleman CL, Bond CM (2016) Defining feasibility and pilot studies in preparation for randomised controlled trials: development of a conceptual framework. PLoS ONE 11(3):e0150205. 10.1371/journal.pone.0150205 26978655 10.1371/journal.pone.0150205 PMC4792418

[CR22] Elliott SK, Drummond M (2015) The (limited) impact of sport policy on parental behaviour in youth sport: a qualitative inquiry in junior Australian football. Int J Sport Policy Politics 7(4):519–530. 10.1080/19406940.2014.971850

[CR23] Elliott SK, Drummond MJN (2017a) Parents in youth sport: what happens after the game? Sport Educ Soc 22(3):391–406. 10.1080/13573322.2015.1036233

[CR24] Elliott SK, Drummond MJN (2017b) During play, the break, and the drive home: the meaning of parental verbal behaviour in youth sport. Leisure Stud 36(5):645–656. 10.1080/13573322.2015.1036233

[CR25] Furusa MG, Knight CJ, Hill DM (2021) Parental involvement and children’s enjoyment in sport. Qualitative Res Sport Exerc Health 13(6):936–954. 10.1080/2159676X.2020.1803393

[CR26] Gonzalez JC, Klein CC, Barnett ML, Schatz NK, Garoosi T, Chacko A, Fabiano GA (2023) Intervention and implementation characteristics to enhance father engagement: a systematic review of parenting interventions. Clin Child Fam Psychol Rev 26(2):445–458. 10.1007/s10567-023-00430-x 36947287 10.1007/s10567-023-00430-x PMC10031187

[CR27] Goodman R (1997) The strengths and difficulties Questionnaire: a research note. J Child Psychol Psychiatry Allied Discip 38(5):581–586. 10.1111/j.1469-7610.1997.tb01545.x 10.1111/j.1469-7610.1997.tb01545.x 9255702

[CR28] Graupensperger S, Sutcliffe J, Vella SA (2021) Prospective associations between sport participation and indices of mental health across adolescence. J Youth Adolesc 50(7):1450–1463. 10.1007/s10964-021-01416-0 33689103 10.1007/s10964-021-01416-0 PMC8222072

[CR29] Harrington M (2006) Sport and leisure as contexts for fathering in Australian families. Leisure Stud 25(2):165–183. 10.1080/02614360500503265

[CR30] Head KJ, Noar SM, Iannarino NT, Grant Harrington N (2013) Efficacy of text messaging-based interventions for health promotion: a meta-analysis. Soc Sci Med 97:41–48. 10.1016/j.socscimed.2013.08.003 24161087 10.1016/j.socscimed.2013.08.003

[CR10] 10.1080/2159676X.2019.1628806

[CR31] Ingoldsby EM (2010) Review of interventions to improve family engagement and retention in parent and child mental health programs. J Child Fam stud 19(5):629–645. 10.1007/s10826-009-9350-2 20823946 10.1007/s10826-009-9350-2 PMC2930770

[CR32] Jewell C, Wittkowski A, Pratt D (2022) The impact of parent-only interventions on child anxiety: a systematic review and meta-analysis. J Affect Disord 309:324–349. 10.1016/j.jad.2022.04.082 35460744 10.1016/j.jad.2022.04.082

[CR35] Knight CJ, Neely KC, Holt NL (2011) Parental behaviours in team sports: how do female athletes want parents to behave? J Appl Sport Psychol 23(1):76–92. 10.1080/10413200.2010.525589

[CR34] Knight CJ, Dorsch TE, Osai KV, Haderlie KL, Sellars PA (2016) Influences on parental involvement in youth sport. Sport Exerc Perform Psychol 5(2):161–178. 10.1037/spy0000053

[CR33] Knight CJ, Berrow SR, Harwood CG (2017) Parenting in sport. Curr Opin Psychol 16:93–97. 10.1016/j.copsyc.2017.03.011 28813364 10.1016/j.copsyc.2017.03.011

[CR36] Lee M, MacLean S (1997) Sources of parental pressure among age group swimmers. Eur J Phys Educ 2(2):167–177. 10.1080/1740898970020204

[CR37] Little RJA (1988) A test of missing completely at random for multivariate data with missing values. J Am Stat Assoc 83(404):1198–1202. 10.1080/01621459.1988.10478722

[CR4] Mallett CJ, Sanders MR, Dittman CK, Kirby JN, Rynne SB (2024) Understanding parenting behavior in junior rugby league in Australia. J Child Fam Stud 33:271–287. 10.1007/s10826-023-02744-4

[CR38] Mossman GJ, Cronin LD (2019) Life skills development and enjoyment in youth soccer: the importance of parental behaviours. J Sports Sci 37(8):850–856. 10.1080/02640414.2018.1530580 30332918 10.1080/02640414.2018.1530580

[CR39] Moulds K, Galloway S, Abbott S, Cobley SP (2022) Youth sport dropout according to the process-person-context-time model: a systematic review. Int Rev Sport Exerc Psychol 1–42. 10.1080/1750984X.2021.2012817

[CR40] Murray KW, Woodruff K, Moon C, Finney C (2015) Using text messaging to improve attendance and completion in a parent training program. J Child Fam stud 24(10):3107–3116. 10.1007/s10826-015-0115-9

[CR41] Mytton J, Ingram J, Manns S, Thomas J (2014) Facilitators and barriers to engagement in parenting programs: a qualitative systematic review. Health Educ Behav 41(2):127–137. 10.1177/1090198113485755 23640123 10.1177/1090198113485755

[CR42] Newby JL, Klein RG (2014) Competitiveness reconceptualised: psychometric development of the competitiveness orientation measure as a unified measure of trait competitiveness. Psychol Record 64(4):879–895. 10.1007/s40732-014-0083-2

[CR44] O’Rourke DJ, Smith RE, Smoll FL, Cumming SP (2011) Trait anxiety in young athletes as a function of parental pressure and motivational climate: is parental pressure always harmful? J Appl Sport Psychol 23(4):398–412. 10.1080/10413200.2011.552089

[CR43] Omli J, Wiese-Bjornstal DM (2011) Kids speak: Preferred parental behaviour at youth sport events. Res Q Exerc Sport 82(4):702–711. 10.1080/02701367.2011.10599807 22276412 10.1080/02701367.2011.10599807

[CR45] Pluhar E, McCracken C, Griffith KL, Christino MA, Sugimoto D, Meehan WP 3rd (2019) Team sport athletes may be less likely to suffer anxiety or depression than individual sport athletes. J Sports Sci Med 18(3):490–496. https://pubmed.ncbi.nlm.nih.gov/31427871/31427871 PMC6683619

[CR46] Reitman D, Currier RO, Hupp SD, Rhode PC, Murphy MA, O’Callaghan PM (2001) Psychometric characteristics of the parenting scale in a Head Start population. J Clin Child Psychol 30(4):514–524. 10.1207/S15374424JCCP3004_08 11708239 10.1207/S15374424JCCP3004_08

[CR47] Roberts GC, Treasure DC, Balague G (1998) Achievement goals in sport: the development and validation of the perception of Success Questionnaire. J Sports Sci 16(4):337–347. 10.1080/02640419808559362 9663958 10.1080/02640419808559362

[CR48] Ryan Dunn C, Dorsch TE, King MQ, Rothlisberger KJ (2016) The impact of family financial investment on perceived parent pressure and child enjoyment and commitment in organised youth sport. Fam Relat 65(2):287–299. 10.1111/fare.12193

[CR49] Sánchez-Miguel PA, Leo FM, Sánchez-Oliva D, Amado D, García-Calvo T (2013) The importance of parents’ behaviour in their children’s enjoyment and amotivation in sports. J Hum Kinetics 36(1):169–177. 10.2478/hukin-2013-0017 10.2478/hukin-2013-0017 PMC3661888 23717366

[CR67] Sanders MR, Baker S, Turner KMT (2012) A randomised controlled trial evaluating the efficacy of Triple P Online with parents of children with early-onset conduct problems. Behav Res Ther 50(11): 675–684. 10.1016/j.brat.2012.07.004 10.1016/j.brat.2012.07.004 22982082

[CR3] Sanders MR, Dittman CK, Mallett CJ, Rynne S (2024) Lessons from the field. Toward evidence‐based parenting support to promote positive parenting in children’s sport. Fam Relat 73(3):2032–2041. 10.1111/fare.12990

[CR53] Sanders MR, Mazzucchelli TG (2013) The promotion of self-regulation through parenting interventions. Clin Child Fam Psychol Rev 16(1):1–17. 10.1007/s10567-013-0129-z 23397366 10.1007/s10567-013-0129-z

[CR51] Sanders MR, Dittman CK, Farruggia SP, Keown LJ (2014a) A comparison of online versus workbook delivery of a self-help positive parenting program. J Prim Prev 35(3):125–133. 10.1007/s10935-014-0339-2 24500106 10.1007/s10935-014-0339-2

[CR52] Sanders MR, Kirby JN, Tellegen CL, Day JJ (2014b) The Triple P-Positive Parenting Program: a systematic review and meta-analysis of a multi-level system of parenting support. Clin Psychol Rev 34(4):337–357. 10.1016/j.cpr.2014.04.003 24842549 10.1016/j.cpr.2014.04.003

[CR50] Sanders MR (2023) The Triple P system of evidence-based parenting support: past, present, and future directions. Clin Child Family Psychol Rev 26(4):880–903. 10.1007/s10567-023-00441-8 10.1007/s10567-023-00441-8 PMC10640495 37432507

[CR55] Smoll FL, Smith RE, Cumming SP (2007) Effects of coach and parent training on performance anxiety in young athletes: a systemic approach. J Youth Development: Bridging Res Pract 2(1):19–36

[CR56] Strandbu Å, Stefansen K, Smette I, Sandvik MR (2019) Young people’s experiences of parental involvement in youth sport. Sport Educ Soc 24(1):66–77. 10.1080/13573322.2017.1323200

[CR57] Sutcliffe JT, Fernandez DK, Kelly PJ, Vella SA (2021) The parental experience in youth sport: a systematic review and qualitative meta-study. Int Rev Sport Exerc Psychol 1–28. 10.1080/1750984X.2021.1998576

[CR58] Tamminen KA, Poucher ZA, Povilaitis V (2017) The car ride home: an interpretive examination of parent–athlete sport conversations. Sport Exerc Perform Psychol 6(4):325–339. 10.1037/spy0000093

[CR59] Telford RM, Telford RD, Cochrane T, Cunningham RB, Olive LS, Davey R (2016) The influence of sport club participation on physical activity, fitness and body fat during childhood and adolescence: the LOOK Longitudinal Study. J Sci Med Sport 19(5):400–406. 10.1016/j.jsams.2015.04.008 26111721 10.1016/j.jsams.2015.04.008

[CR60] Thrower SN, Harwood CG, Spray CM (2017) Educating and supporting tennis parents: an action research study. Qualitative Res Sport Exerc Health 9(5):600–618. 10.1080/2159676X.2017.1341947

[CR61] Thrower SN, Harwood CG, Spray CM (2019) Educating and supporting tennis parents using web-based delivery methods: a novel online education program. J Appl Sport Psychol 31(3):303–323. 10.1080/10413200.2018.1433250

[CR62] Trost SG, Sallis JF, Pate RR, Freedson PS, Taylor WC, Dowda M (2003) Evaluating a model of parental influence on youth physical activity. Am J Prev Med 25(4):277–282. 10.1016/s0749-3797(03)00217-4 14580627 10.1016/s0749-3797(03)00217-4

[CR63] Turner KMT, Sanders MR (2011) Triple P online [Interactive internet program]. Triple P International Pty. Ltd. www.triplep.net, Brisbane, QLD, Australia

[CR64] Vella SA, Cliff DP, Magee CA, Okely AD (2015) Associations between sports participation and psychological difficulties during childhood: a two-year follow up. J Sci Med Sport 18(3):304–309. 10.1016/j.jsams.2014.05.006 24908361 10.1016/j.jsams.2014.05.006

[CR65] Wilson OWA, Whatman C, Walters S, Keung S, Enari D, Rogers A, Millar S-K, Ferkins L, Hinckson E, Hapeta J, Sam M, Richards J (2022) The value of sport: Wellbeing benefits of sport participation during adolescence. Int J Environ Res Public Health 19(14). 10.3390/ijerph19148579 10.3390/ijerph19148579 PMC9324252 35886430

[CR66] Zuckerman SL, Tang AR, Richard KE, Grisham CJ, Kuhn AW, Bonfield CM, Yengo-Kahn AM (2021) The behavioural, psychological, and social impacts of team sports: a systematic review and meta-analysis. Physician Sports Med 49(3):246–261. 10.1080/00913847.2020.1850152 10.1080/00913847.2020.1850152 33196337

