# Supplementary material for: Play Well Triple P: Developing and Evaluating a Program to Promote Positive Parental Involvement in Junior Sport
Source: Child Psychiatry Hum Dev. 2024 Jun 18;57(2):516–32. doi: 10.1007/s10578-024-01725-y (PMC13128719; doi:10.1007/s10578-024-01725-y)
Supplement: Supplementary file 1 — Supplementary Material 1 [file 10578_2024_1725_MOESM1_ESM.docx]

**Supplementary Table 1** Overview of Play Well Triple P online content^a^

| Segment | Title | Topics covered |
| --- | --- | --- |
| Segment 1 | Why is sport important for children? | Health, social and psychological benefits of sport |
| Segment 2 | Role of parents and Common parent traps | The important role of parents in sport  Common parent traps   - Kids watch others (being a poor role model) - Negativity and criticism - Ignoring positive behaviour - Too much focus on winning (unhelpful beliefs) - Too high expectations (unrealistic expectations) - Pressure to perform - Difficulty managing emotions - Outside influences on children’s behaviour |
| Segment 3 | Tips for being a positive sporting parent | - Be a good role model - Keep thoughts and emotions in check - Be an interested, tuned in observer - Give support and encouragement - Be a positive and respectful supporter of the game - Give helpful feedback at a time that is right - Be an active family - Be prepared and reliable |
| Segment 4 | Wrap up and Call to action | Summary  Accessing further parenting support  Call to action |

*Note.* ^a^ The same topics were covered in both the prototype (Study 1) and pilot versions (Study 2) of Play Well Triple P. However, the video segments were structured differently in the prototype version such that there was more video content, and that content was split across a larger number of video segments.
